# Oestradiol enhances tumour regression induced by B7-1/IL-2 adenoviral gene transfer in a murine model of breast cancer

**DOI:** 10.1038/sj.bjc.6601099

**Published:** 2003-07-15

**Authors:** C Dabrosin, K Palmer, J Gauldie

**Affiliations:** 1 Department of Pathology and Molecular Medicine, Centre for Gene Therapeutics, McMaster University, Hamilton, Ontario, Canada L8N 3Z5

**Keywords:** immunotherapy, mammary cancer, polyomavirus, sex steroids, adenovirus

## Abstract

The majority of breast cancers are oestrogen dependent and although current treatment strategies have improved, approximately 50% of the patients will develop metastasis. New treatments that result in long-term systemic immunity are therefore being developed. We have previously shown that adenoviral gene transfer of B7-1/IL-2 to murine breast cancer induces a high rate of complete tumour regression and systemic immunity. Since oestrogens not only affect breast cancer but also have been shown to modulate immune function and secretion of immune-regulatory cytokines, we explored whether administration of oestradiol altered the immune response induced by an adenoviral vector expressing B7-1/IL-2. An oestrogen-dependent murine breast cancer tumour was used in ovariectomised mice, supplemented either oestradiol or placebo. We report the somewhat unexpected finding that intratumoral injection of adenovirus expressing B7-1/IL-2 induces complete tumour regression in 76% of oestradiol-supplemented mice, while only 18% of the tumours regressed in the oestrogen-depleted group. Cured mice in both groups exhibited a similar CTL response against the tumour antigen. However, intratumoral IFN-*γ* levels, 2 days after B7-1/IL-2 injection, were significantly higher in mice treated with oestradiol compared to placebo. This may be one mechanism explaining the higher response rate of tumours in oestradiol-replenished mice.

Breast cancer accounts for almost 30% of all cancer cases in women in the Western World and the incidence of the disease is still increasing (Ries *et al*, 2001). Oestrogen exposure plays an important role in the risk of developing breast cancer. Prolonged exposure of sex steroids increase the risk, while surgically induced menopause by bilateral ovariectomy reduces the risk of the disease (Schairer *et al*, 1997; Rossouw *et al*, 2002). Approximately 70% of all breast cancers express the oestrogen receptor (ER), and treatments designed to block oestrogen effects in the breast are important options in the clinic (Lapidus *et al*, 1998). However, 50% of women with primary tumours will develop metastasis and eventually die from the disease (Klijn *et al*, 1992). Therefore, new treatments such as immunogene therapy that results in long-term systemic immunity, which may prevent metastatic disease, are being developed.

Gene therapy enhancing the immune response against cancer cells includes immunisation, introduction of cytokines and expression of T-cell costimulatory molecules. Presentation of antigen to the T-cell receptor is necessary for the initiation of an immune response. However, additional molecules expressed on antigen-presenting cells deliver essential costimulatory signals and in the absence of costimulation T-cell anergy is developed. The B7 costimulatory molecules B7-1 (CD80) and B7-2 (CD86) are required for full activation of T cells. The B7 molecules are members of the immunoglobulin (Ig) superfamily and are ligands for CD28 and CTLA-4 counter-receptors on T cells (Brunet *et al*, 1987; Freeman *et al*, 1989, 1991; Linsley *et al*, 1990). Ligation of CD28 with B7-1 or B7-2 results in upregulation of the IL-2 receptor *α*, *β* and *γ* chains (Cerdan *et al*, 1992, 1995), increased IL-2 gene transcription (Fraser *et al*, 1991), upregulation of CTLA-4 mRNA levels (Lindsten *et al*, 1993), cytokine secretion and T-cell proliferation (Linsley *et al*, 1991; Freeman *et al*, 1993; Chambers, 2001). Expression of B7 family members in murine tumour models has been shown to activate CD8+ T cells and/or CD4+ T cells against tumour cells (Freeman *et al*, 1991, 1993; Townsend and Allison, 1993). Administration of IL-2 has been shown to promote antitumour immunity, presumably by alleviating the anergic block seen in T cells in some tumour models and thereby preventing the onset of anergy (Schwartz, 1990). We have previously shown that an adenovirus vector expressing human IL-2 causes complete tumour regression in approximately 20–40% of the cases, whereas a simultaneous expression of B7-1 and IL-2 in a single vector have a much higher success rate, >90% (Emtage *et al*, 1998).

There are known gender differences in the immune response. Women have an increased incidence of autoimmune diseases and a better outcome after sepsis or trauma and oestrogens have been shown to modulate immune function (Ansar Ahmed *et al*, 1985; Schroder *et al*, 1998). Oral contraceptives and pregnancy alter the course of many autoimmune diseases in women (Paavonen, 1994). It has also been shown that oestrogens can regulate the expression of IL-2, IL-6, IL-10 and IFN-γ in several cell systems (Gilmore *et al*, 1997; Correale *et al*, 1998; Keck *et al*, 1998; Rogers and Eastell, 1998).

Given the impact oestrogens have on both breast cancer and the immune function, the objective of this study was to explore whether oestradiol altered the immune response induced by intratumoral injection of an adenoviral vector expressing B7-1/IL-2, in a murine oestrogen-dependent model of breast cancer.

In this study, we used ovariectomised mice supplemented with physiologic levels of oestradiol or left oestrogen depleted. We demonstrate that oestradiol supplementation results in markedly enhanced tumour regression and that this is associated with raised levels of intratumoral IFN-*γ*.

## MATERIALS AND METHODS

### Animals and ovariectomy of mice

FVB/n mice (6–8 weeks old) were purchased from Charles River (Troy, NY, USA). They were housed in pathogen-free conditions with a light/dark cycle of 12/12 h and fed with rodent chow and water *ad libitum*. The McMaster University animal ethics research board approved all animal work, which was conducted according to the UKCCCR guidelines (Workman *et al*, 1998).

Mice were anaesthetised with isofluorane (Abbot), ovariectomised (OVX) and 3-mm pellets containing 17 *β*-oestradiol (E2), 0.18 mg per 60 day release, or placebo pellets (Innovative Research of America, Sarasota, FL, USA) were implanted subcutaneously (s.c.) in the animal's back 7 days before tumour induction. The pellets provide continuous release of E2 at serum concentrations of 150–250 pM, confirmed by serum analysis assayed by Abbot Axsym Systems (Abbot, IL, USA), which is in the range of physiologic levels seen in mice during the oestrous cycle (Loeb and Qiumby, 1987).

### Polyoma middle T tumour model

Tumour cells were derived from a transgenic mouse strain expressing polyoma middle T (PyMT) antigen under the control of the mouse mammary tumour virus (MMTV) long-terminal repeat (Guy *et al*, 1992). These mice develop spontaneous adenocarcinomas of all mammary epithelium by 8–10 weeks of age. These tumours were excised, minced and incubated at 37°C with gentle stirring in collagenase/dispase solution (25 mg collagenase 250 mg dispase, Roche, in 100 ml phosphate-buffered saline (PBS) to generate a single-cell suspension, stored at −70°C in foetal bovine serum with 10% DMSO added. Thawed cells were placed in complete F-11 medium and were cultured until they reached confluence, usually 4–6 days. Thereafter, 1 × 10^6^ cells in 200 *μ*l PBS were injected s.c. into the right hind flank of a syngenic female FVB/n host. Tumour volume was determined by measuring length, width, and depth of the tumour weekly using a caliper.

### Recombinant adenoviruses

The vector expressing both human IL-2, known to be active across species, and the murine costimulatory molecule B7-1(AdB7-1/IL-2), and the control vector Ad*dl*70-3 were constructed as previously described (Bett *et al*, 1994; Emtage *et al*, 1998).

### Intratumour vector administration and rechallenge of tumour-free mice

Following tumour formation (tumour volume <250 mm^3^) animals were injected intratumorally with 1 × 10^9^ PFU of AdB7-1/IL-2 or control Ad*dl*70-3 vector diluted (50 *μ*l) in sterile endotoxin-free PBS and tumour growth determined weekly. In animals that showed complete regression of primary PyMT tumours, mice were rechallenged at 90 days with PyMT tumour cells by subcutaneous injection of 10^6^ tumour cells on the opposite flank.

### Cytotoxic T-cell assay

Specific PyMT antigen cytotoxic T-cell killing was measured as previously described (Addison *et al*, 1998; Palmer *et al*, 2001). Briefly, splenocytes were obtained from mice whose tumours had regressed and had also resisted rechallenge and were restimulated for 5 days by coculture with irradiated 516MT3 cells that express the PyMT antigen. Cytotoxicity was determined by coculture with 516MT3 or PTO516 (PyMT antigen-negative cells) target cells, labelled with ^51^Cr. The percent specific lysis was calculated as follows: 100 × (experimental c.p.m. −spontaneous c.p.m.) (maximal c.p.m. −spontaneous c.p.m.).

### Quantification of murine IFN-γ in tumour tissue and plasma

Tumours were removed, snap frozen in liquid nitrogen, and weighed 2 and 10 days after adenovirus administration. Tumours were homogenised in PBS containing 100 *μ*M phenylmethylsulphonyl fluoride, centrifuged and the supernatants were stored at −70°C for subsequent analysis. Plasma was collected by cardiac puncture. IFN-*γ* was measured using an ELISA kit (R&D Systems, Minneapolis, MN, USA). The sensitivity was 2 pg ml^−1^. All samples were analysed in duplicates and given results are the means of each duplicate.

### Statistics

Data are expressed as mean±s.e. m. All given values represent the mean of the duplicate analysis of each sample. Student's *t*-test and Fisher's exact test was used as appropriate. A *P*<0.05 was considered as statistically significant.

## RESULTS

### Enhanced tumour regression by oestradiol after intratumoral administration AdB7-1/IL-2

We have recently characterised the oestrogenic phenotype of the PyMT model used in this study. These tumours express the ER and E2 stimulates tumour cell proliferation *in vitro* and tumour growth *in vivo* (Dabrosin *et al*, 2003). Moreover, E2 enhances tumour angiogenesis *in vivo* (Dabrosin *et al*, 2003). The recombinant adenovirus vector expressing murine B7-1 and human IL-2 has previously been characterised and expression of both molecules have been confirmed (Emtage *et al*, 1998).

Administration of 1 × 10^9^ PFU of the B7-1/IL-2 vector resulted in complete tumour regression within 3 weeks in 76% of OVX+E2-treated animals, while only 18% of the placebo animals were cured. In the E2-treated group, all tumours showed either partial or total regression with an overall response rate of 100%. Partial regression was defined as stasis or decrease in tumour volume 1 week after the administration of a vector, as compared with the original tumour volume before treatment. In the OVX animals without E2 supplement 60% of the tumours had a delayed tumour growth without cure and 23% of the tumours failed to respond at all. None of the tumours in OVX or OVX+E2 animals treated with the control vector Ad*dl*70-3 had any response and none of the mice were cured, a result that we previously have reported in normal cycling mice (Emtage *et al*, 1998) (Table 1

**Table 1:** Summary of responses of PyMT tumours following intratumoral injection of AdB7-1/IL-2 and Ad*dl*70-3 in ovariectomised mice treated with placebo (OVX) or oestradiol (E2)

**Treatment**	**No response (%)**	**Partial response (%)**	**Total regression (%)**
OVX control Ad*dl*70-3	5/5 (100)	0/5 (0)	0/5 (0)
OVX+E2 control Ad*dl*70-3	5/5 (100)	0/5 (0)	0/5 (0)
OVX AdB7-1/IL-2	5/22 (23)	13/22 (59)	4/22 (18)
OVX+E2 AdB7-1/IL-2	0/17 (0)	4/17 (24)	13/17 (76)*

Partial response refers to tumours that have undergone partial regression followed by regrowth, or a delay in growth; no response refers to tumours that have continued to grow at a rate comparable to controls.

**Figure 1:**
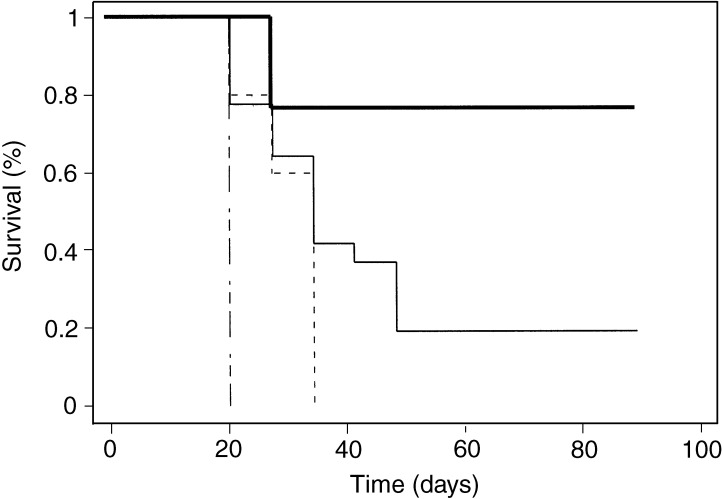
Long-term survival of ovariectomised (OVX) FVB/n mice treated with oestradiol (E2) or placebo bearing small PyMT tumours following treatment with recombinant adenovirus expressing B7-1/IL-2 or control virus Ad*dl*70-3 at a dose of 1 × 10^9^ PFU. OVX+E2-treated tumours (228±60 mm^3^) injected with AdB7-1/IL-2 (*n*=17), OVX+placebo (160±28 mm^3^) injected with AdB7-1/IL-2 (*n*=22). Tumours from both groups (OVX and OVX+E2, n=10–11 in each group) injected with the control virus Ad*dl*70-3, all succumbed to tumour growth. (**——**) AdB7-1/IL-2 OVX+E2, (——) AdB7-1/IL-2 OVX+placebo, (— - –) Ad*dl*70-3 OVX+E2, (**– – – –**) Addl70-3 OVX+placebo.

* *P*<0.005 compared to OVX.

). The survival data of all animals in this study are presented in Figure 1

. As previously shown, the cytokines delivered intratumorally did mediate an effective immune response and tumour regression, whereas systemic delivered cytokines fail to induce any tumour response (Addison *et al*, 1995). The tumour size, 160±28 mm^3^ for placebo tumours and 228±60 mm^3^ E2-treated tumours, was in the range previously described by our lab using B7-1/IL-2 and other cytokines delivered to PyMT tumours by adenoviral vectors (Addison *et al*, 1998; Emtage *et al*, 1998; Palmer *et al*, 2001).

**Figure 2:**
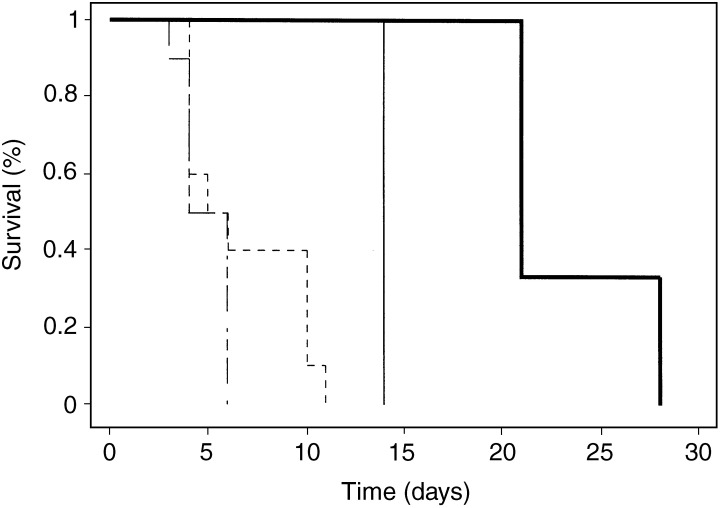
Survival of mice following intratumoral injection of large tumours with recombinant adenovirus expressing B7-1/IL-2. Mice treated as described in Figure 1. OVX+E2 (*n*=3, initial size 1150±132 mm^3^) and OVX+placebo (*n*=3, 1170±60 mm^3^) were injected with 1 × 10^9^ PFU of AdB7-1/IL-2. Control animals were injected with Ad*dl*70-3, 1 × 10^9^ PFU (*n*=10 in each group with similar tumour sizes, 1230±150 mm^3^, as the treated tumours). (**——**) AdB7-1/IL-2 OVX+E2, (——) AdB7-1/IL-2 OVX+placebo, (**— - –**) Ad*dl*70-3 OVX+E2, (**– – – –**) Ad*dl*70-3 OVX+placebo.

A few tumours in each group were allowed to grow considerably larger before AdB7-1/IL-2 injection, OVX+placebo 1170±60 mm^3^ and E2 tumours 1150±132 mm^3^. In these larger tumours, a partial regression was induced by AdB7-1/IL-2 delivery to E2-treated mice, while placebo mice continued to grow without delay (Figure 2

).

### Oestradiol increased intratumoral IFN-*γ* production induced by AdB7-1/IL-2

**Figure 3:**
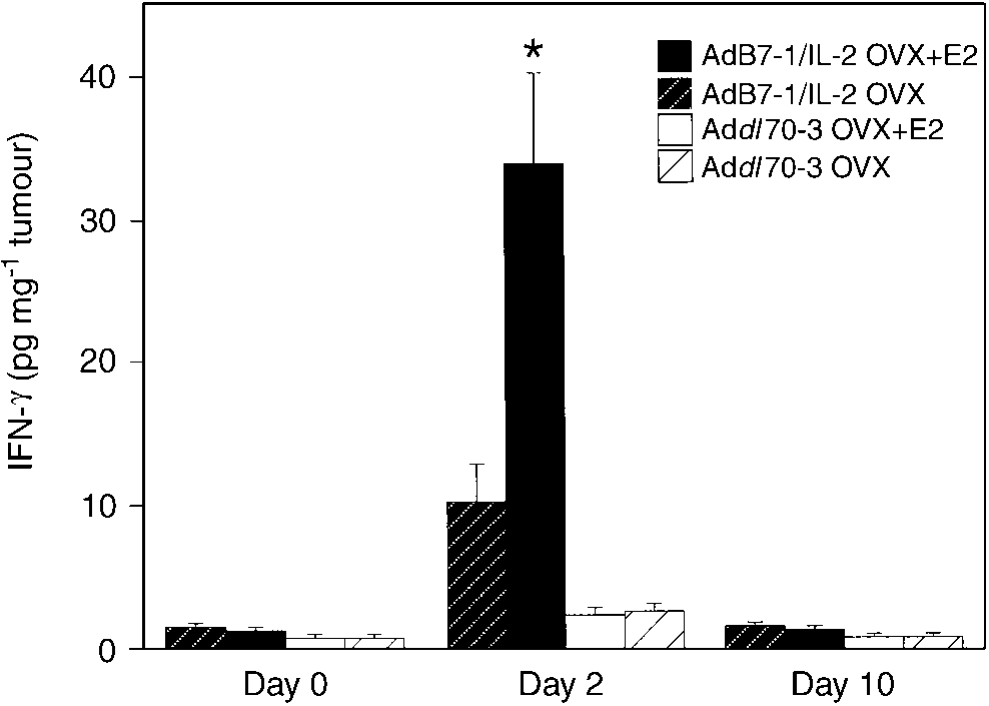
Oestradiol increased local IFN-*γ* levels 2 days following treatment with AdB-7/IL-2. Mice treated as described in Figure 1. IFN-*γ* was measured in tumour homogenate and normalised to the mass of tumour. Oestradiol tumours treated with AdB7-1/IL-2 had significantly higher levels of IFN-*γ* on day 2, OVX+E2 *n*=4, *vs* OVX+placebo *n*=3, ^*^P<0.05. There was no difference between the groups on days 0 and 10.

At 2 days after intratumoral administration of AdB7-1/IL-2, both E2-exposed tumours and placebo tumours had increased levels of IFN-*γ* compared with tumours injected with the control vector Ad*dl*70-3, 24±0.6 pg mg^−1^ tumour in AdB7-1/IL-2 *vs* 3±0.04 pg mg^−1^ tumour with the control vector. However, tumours from E2-treated mice had significantly higher levels of IFN-*γ* compared with placebo-treated mice, 34±0.6 pg mg^−1^ tumour *vs* 10±0.3 pg mg^−1^ tumour, *P*<0.01, *n*=3–4 in each group (Figure 3

). Tumour levels of IFN-*γ* and the magnitude of the increase in responders compared to nonresponders are in line with the previous work (Bramson *et al*, 1996). The increased levels of IFN-γ were localised to the tumour since both groups had similar plasma levels of IFN-*γ*, 52±5 pg ml^−1^. At 10 days after vector administration, the tumour levels of IFN-*γ* in both groups had decreased to 1.4±0.02 pg mg^−1^ tumour (Figure 3). There was no difference between E2-treated and placebo mice in the control group (Figure 3).

### Generation of long-term systemic immunity to PyMT tumour cell rechallenge

Mice that had undergone complete regression of the PyMT tumours after treatment with AdB7-1/IL-2 were rechallenged with a second inoculation of 1 × 109 PyMT cells 90 days after initial vector administration, on the left hind flank. None of the challenged mice developed tumours and all remained tumour free during the observation period (90 days). Naive mice simultaneously injected with the same tumour cell suspension developed tumours within 21 days as expected. These results suggest that the cured mice in either E2 or placebo group developed long-term antitumour immunity after the AdB7-1/IL-2 treatment. These results are in line with our previous data showing that cured mice develop long-term immunity regardless of the cytokine vector used, although the different vectors exhibit higher or lower effectiveness in inducing complete tumour regression (Emtage *et al*, 1998; Palmer *et al*, 2001).

### CTL activity in cured mice

**Figure 4:**
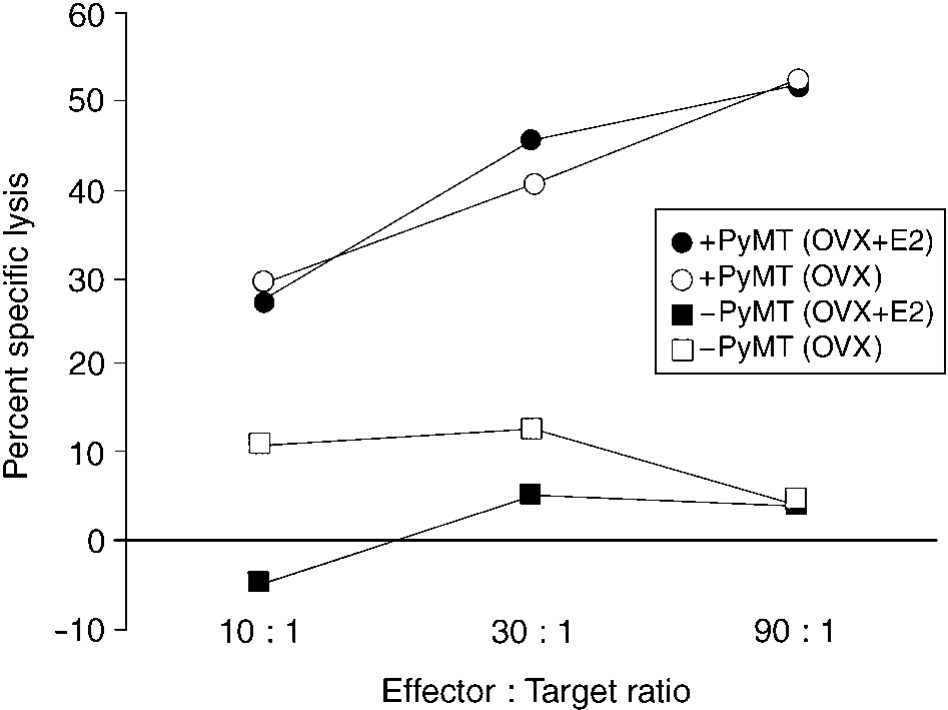
Cytotoxic T lymphocyte activity in mice after total tumour regression induced by AdB71/IL-2 treatment. Spleens were removed from mice, which had undergone complete regression of PyMT breast cancer tumours and not developed tumours after having been rechallenged with fresh tumour cells. Splenocytes were used in a ^51^Cr release assay against target cells (516MT3) with or without the PyMT antigen, PT0516 (-PyMT).

To determine whether oestradiol supplement may affect the development of a tumour-specific CTL response, cured mice in both the E2 and placebo group were killed and their spleens removed 6 weeks following PyMT rechallenge. Splenocytes prepared from either group demonstrated a 50% specific lysis of 516MT3 cells, expressing the PyMT antigen, at an effector to target ratio of 90 : 1 (Figure 4

). Minimal background was observed. This suggests the presence of significant numbers of effector cells capable of killing cells expressing the PyMT tumour antigen in both treatment groups. Splenocytes from control mice did not exhibit any tumour-specific CTL activity as previously shown (Emtage *et al*, 1998).

## DISCUSSION

In this study, we demonstrated that oestradiol, at physiologic concentrations, enhances the immune response by intratumoral adenoviral gene delivery of B7-1/IL-2 in a murine model of breast cancer in which the growth rate is increased by oestrogen. This difference was not due to an inability of oestrogen-depleted mice to induce a CTL response since cured mice from both groups exhibited similar CTL activity. However, we show that oestradiol increased the local IFN-γ response after delivery of AdB7-IL-2, which may be one mechanism to explain the higher response rate in the oestradiol-supplemented mice.

Distinct patterns of cytokine expression are associated with the development of TH1 or TH2 immune responses (Mosmann and Coffman, 1989; Mosmann and Sad, 1996). Development of CTL response involves IL-2 and IFNγ, while humoral responses are associated with IL-4, IL-6 and IL-10. IFN-γ is important for the development of a potent antitumour response (Ogawa *et al*, 1998). Our previous studies with the PyMT tumour model have shown that lymphocytes isolated from cured mice exhibited raised levels of IFN-*γ* indicating that treatment with Ad vectors induces cytokines and costimulatory molecules, which induce TH1-mediated antitumour responses (Bramson *et al*, 1996; Addison *et al*, 1998). Moreover, IFN-*γ* affects tumour cells to be more susceptible to T-cell-mediated killing (Gerrard *et al*, 1988) and upregulates the chemokines IP-10 (interferon-inducibleprotein) and MIG (monokine induced by gamma interferon) resulting in decreased angiogenesis (Angiolillo *et al*, 1995; Palmer *et al*, 2001). IFN-*γ* also appears to sensitise human breast cancer cells to undergo apoptosis (Ruiz-Ruiz *et al*, 2000).

It has been suggested that females produce stronger immune reactions than males (Ansar Ahmed *et al*, 1985). Autoimmune diseases such as multiple sclerosis and rheumatoid arthritis are more frequent in women and patients go into a clinical remission during pregnancy and exacerbate postpartum suggesting that the immune response differs with various levels of oestrogen (Ostensen *et al*, 1983; Confavreux *et al*, 1998). Oestradiol has been found to modulate the secretion of cytokines from human CD4+ cells, at physiological levels oestradiol increased IFN-*γ* production with no effect on IL-10 secretion (Gilmore *et al*, 1997). However, at pharmacological levels or levels seen in pregnancy, the IL-10 levels increased significantly, while IFN-*γ* remained at the same levels seen with a low dose of oestradiol (Gilmore *et al*, 1997). Moreover, lymphocytes from female mice produce higher levels of IFN-*γ* after immune stimulation than those from males and an oestrogen-responsive element in the IFN-*γ* promoter in murine spleen cells has been found (McFarland and Bigley, 1989; Fox *et al*, 1991). In addition, both oestrogen and B7 molecules have the ability to activate CD8+ cytotoxic cells even in the absence of CD4+ (Stimson, 1988; Van Gool *et al*, 1996). However, other mechanisms may also contribute in the higher response of oestradiol treated tumours. We have recently shown that oestradiol-treated PyMT tumours used in this experiment have a higher microvessel count compared with tumours at the same size grown in OVX mice with placebo supplement (Dabrosin *et al*, 2003). This could affect the access of the tumour cells to the immune cells, that is, oestradiol-treated tumours have higher blood flow and are thereby more available to the immune system.

None of the larger established tumours underwent complete regression, but the tumours from oestradiol-treated mice showed a partial response and delayed tumour growth. This suggests that tumour size is a predictive factor for the success rate of immune gene therapy and that a dose adjustment or other adjuvant therapies are needed for treatment of heavy tumour burden. This is in line with current clinical principles where tumour size is a strong predictive factor for overall survival and relapse-free interval and larger tumours are treated with surgery in combination radiotherapy and/or chemotherapy (Linderholm *et al*, 2000; Bergh *et al*, 2001).

The results in our study are intriguing and somewhat unexpected since the cornerstone of treatment of oestrogen-dependent breast cancer is antioestrogen hormonal therapy. However, the action of oestrogen in breast cancer is complex, poorly understood and contradictory. While oestrogen stimulates tumour cell growth *in vitro* at low doses, it inhibits proliferation at high levels (Lippman *et al*, 1976). *In vivo*, breast tumours may maintain high local levels of oestrogen independent on the overall circulating concentration suggesting that added exogenous oestrogen has very little effect on the local tumour environment (Blankenstein *et al*, 1992). Oestrogens have the ability to affect tumour cell division, angiogenesis, bone density and the immune system, and the overall result *in vivo* is dependent on the balance between all these effects. Moreover, oestrogen effects are related to the dose and type of hormone used. It is therefore important to use physiologic levels of oestrogen and the naturally occurring E2 in studies of pathogenesis and immune modulation.

We conclude that oestradiol increases the cure rate of oestrogen-enhanced murine breast cancer treated with an intratumoral adenoviral vector expressing B7-1/IL-2. Oestradiol treatment increased the IFN-*γ* levels in tumour tissue, measured 2 days after tumour injection, which may be involved in the mechanisms leading to the higher response rate in these mice. Future studies will determine whether these data are valid for human breast cancer and how the partial oestrogen agonist tamoxifen, the most commonly used antioestrogen in breast cancer treatment, affects immune-stimulatory breast cancer therapies.
